# A Structural Approach to the Strength Evaluation of Linear Chalcogen Bonds

**DOI:** 10.3390/molecules28073133

**Published:** 2023-03-31

**Authors:** Maria Carla Aragoni, Massimiliano Arca, Vito Lippolis, Anna Pintus, Yury Torubaev, Enrico Podda

**Affiliations:** 1 Dipartimento di Scienze Chimiche e Geologiche, Università degli Studi di Cagliari, Cittadella Universitaria, S.S. 554-Bivio Sestu, 09042 Monserrato, CA, Italy; marca@unica.it (M.A.); lippolis@unica.it (V.L.); apintus@unica.it (A.P.); enrico.podda@unica.it (E.P.); 2 Department of Chemistry, Ben-Gurion University of the Negev, 84105 Beer-Sheva, Israel; torubaev@bgu.ac.il; 3 Centro Servizi di Ateneo per la Ricerca, Università degli Studi di Cagliari, Cittadella Universitaria, S.S. 554 Bivio Sestu, 09042 Monserrato, CA, Italy

**Keywords:** sulfur, selenium, tellurium, chalcogen bonding, structural data, crystallographic distances

## Abstract

The experimental structural features of chalcogen bonding (ChB) interactions in over 34,000 linear fragments R–Ch⋯A (Ch = S, Se, Te; R = C, N, O, S, Se, Te; A = N, O, S, Se, Te, F, Cl, Br, I) were analyzed. The bond distances d_R–Ch_ and the interaction distances d_Ch⋯A_ were investigated, and the functions δ_R–Ch_ and δ_Ch⋯A_ were introduced to compare the structural data of R–Ch⋯A fragments involving different Ch atoms. The functions δ_R−Ch_ and δ_Ch⋯A_ were calculated by normalizing the differences between the relevant bond d_R–Ch_ and ChB interaction d_Ch⋯A_ distances with respect to the sum of the relevant covalent (*r_cov_R* + *r_cov_Ch*) and the van der Waals (vdW) radii (*r_vdW_Ch* + *r_vdW_A*), respectively. A systematic comparison is presented, highlighting the role of the chalcogen involved, the role of the R atoms covalently bonded to the Ch, and the role of the A species playing the role of chalcogen bond acceptor. Based on the results obtained, an innovative approach is proposed for the evaluation and categorization of the ChB strength based on structural data.

## 1. Introduction

**Scheme 1:**
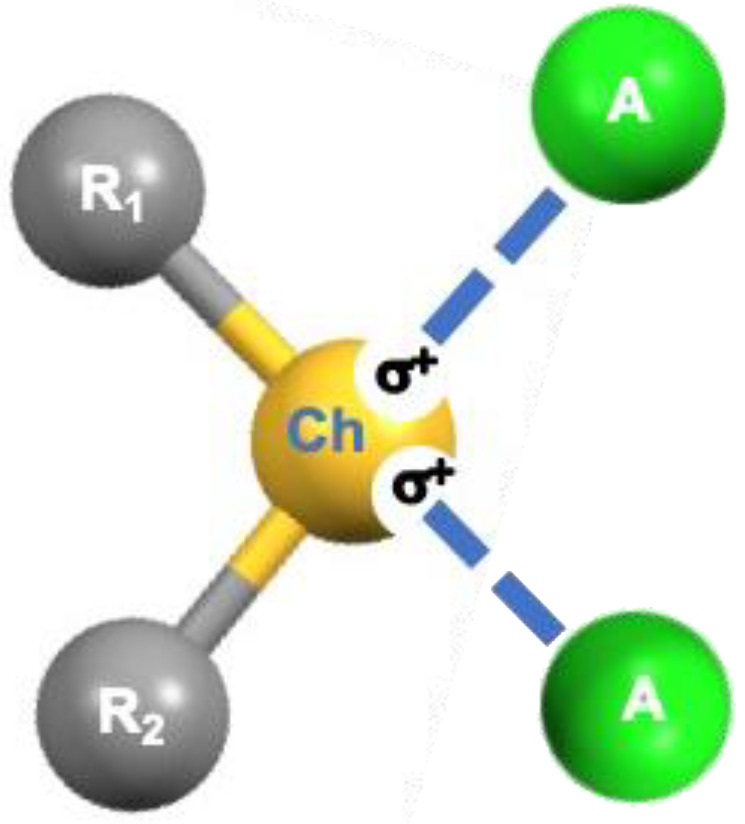
Schematic representation of ChB formation.

Most neutral organic compounds containing chalcogen elements (Ch) belong to the categories of chalcogenones R=Ch, chalcogeno-ethers R−Ch−R, and chalcogen oxides R−(Ch=O)−R. In these compounds, Ch elements feature an anisotropic electron-density distribution defining regions where the electrostatic potential can assume negative and positive values, respectively [1]. Regions of electron density depletion [2] and/or with positive or less negative electrostatic potentials [3], commonly named σ-holes, are usually located opposite to the R−Ch bonds and can interact attractively with electron-rich sites (A) such as atoms and anions bearing a lone pair of electrons (Lp, Scheme 1 and Scheme S1), forming the so-called chalcogen bonds (ChBs).

These interactions involve chalcogen atoms acting as electrophiles and must be distinguished from those in which the Ch atom acts as a nucleophile, such as hydrogen or halogen bonds (HB and HaB, respectively) [4,5,6]. The earliest crystallographic contribution to the understanding of interactions involving chalcogen atoms acting as electrophiles was reported by J.D. Duniz in 1977 [7], who described the dual role played in dialkyl sulfides by the divalent sulfur atom, which undergoes an electrophilic attack approximately perpendicular to the R−S−R plane and a nucleophilic attack along the extension of the S−R covalent bonds. However, interest in these interactions only began to increase after 2007, when a bonding model of ChB was proposed [8]. Currently, in accordance with the recent IUPAC definition [9], in a typical chalcogen-bonded system R–Ch⋯A, ChBs are considered to be highly-directional interactions between an electrophilic region associated with a chalcogen atom in one molecular entity, R–Ch, acting as a Lewis acid electrophilic species (ChB donor; Ch = mainly S, Se, or Te), and a nucleophilic region in another, or the same, molecular entity, A, acting as a Lewis base nucleophilic center (ChB acceptor; A = Lp-bearing atom, anion, π-system, radical, etc.). ChBs can be identified by considering the contacts between Group 16 elements and Lp-bearing atoms in crystalline solids with interatomic distances intermediate between those of typical single bonds and the sum of the van der Waals radii [9,10,11]. It is generally accepted that the strength of the ChB increases (1) with the electron-withdrawing ability of the substituents directly attached to the electron-acceptor Ch atom and (2) with the atomic number of the Ch species [12]. Following the surge of interest in HaB, research on ChB is undergoing a rapid expansion and several recent reviews have covered fundamental aspects and applications of this type of interaction and highlighted examples from the literature [13,14,15,16,17,18,19,20]. Despite the large number of scientific contributions on ChB interactions, a systematic investigation and comparative analysis of the available structural data is still lacking in the literature, as the existing reports generally focus on selected groups of closely related compounds and are often based on theoretical studies using model compounds. In this paper, we present an extended systematic investigation of the structural features of the linear fragments R−Ch⋯A (R−Ch⋯A angle in the range 160–180°; Ch = S, Se, Te; A = N, O, S, Se, Te, F, Cl, Br, and I) retrieved from the Cambridge Structural Database (CSD) where the interatomic distance Ch⋯A is shorter than the sum of the van der Waals radii, as defined by IUPAC [9]. The structural features of ChB are discussed, pointing out the differences that occur when the chalcogen element is varied, and the nature of R and A. It is interesting to note that most of the examined structures were published in papers in which the authors did not identify or mention the presence of a ChB, so the present study covers a large amount of crystallographic data that has never been studied before from this perspective.

## 2. Results and Discussion

Several theoretical approaches have been adopted to interpret ChB interactions, including Natural Bonding Orbitals (NBO) [21], Quantum Theory of Atoms in Molecules (QTAIM) [18,22], Charge Displacement Analysis (CDA) [23], and Energy Decomposition Analysis (EDA) [18]. In general, R–Ch⋯A ChB interactions result from a subtle balance between attractive electrostatic [24,25,26], covalent, and dispersion [27] contributions [28,29], and steric (Pauli) repulsion [28] terms. In addition, ChB interactions can be influenced by cooperative and/or competitive non-covalent interactions of different nature, such as HBs, HaBs, π-stacking interactions, and so forth [18].

The nature of the ChB interactions strongly depends on the interacting Ch and A atoms in the R−Ch⋯A system, with ChB becoming stronger with the polarizability of the chalcogen atom Ch (Te > Se > S). The electron-withdrawing ability of the R substituent in turn increases the interaction energy and shortens the Ch⋯A intermolecular distance [30].

A list of typical ChB donors and acceptors [9] shows that R–Ch donors commonly involved in ChB are chalcogen-containing heterocycles and their fused polycyclic derivatives; chalcogen-anhydrides and their analogues with sulfonyl groups in place of carbonyl ones; and chalcogen-cyanates and chalcogen oxides, in which the R atom directly bonded to the chalcogen is usually either a carbon, a nitrogen, or another chalcogen atom. ChB acceptors are nucleophiles with Lp-bearing atoms and anions, in which the A atom directly involved in ChB is typically either a pnictogen (usually nitrogen), another chalcogen, or a halogen atom. Unsaturated systems in which the ChB acceptor is a multiple bond or an aromatic moiety will not be examined here, and the reader is referred to specific publications for an overview of this topic [31,32,33,34,35].

### 2.1. R–S⋯A Fragments

Among the nearly thirty thousand R–S⋯A fragments (27,763 entries) found in the database (Table S1), about 80% of the hits (21,993 entries) feature a carbon atom directly bonded to the sulfur atom (C−S⋯A; R = C). The remaining 20% are mainly composed of N−S⋯A (9%; R = N) and Ch−S⋯A (10%; R = S) fragments, with a very small contribution coming from all the other systems (1%), which are not included in the following analysis.

**Figure 1:**
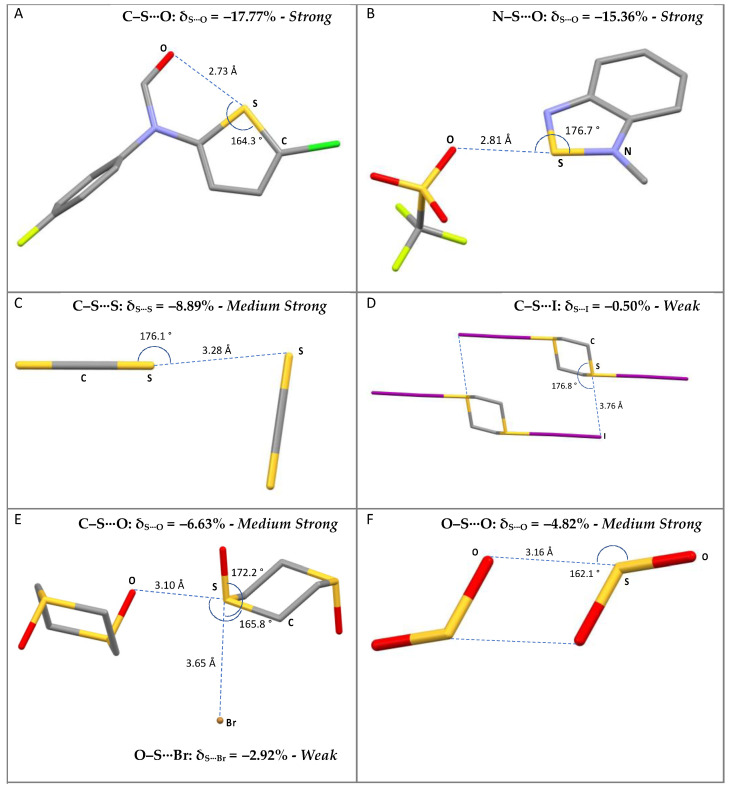
Examples of selected R–S⋯A fragments featuring a chalcogen bond and corresponding CCDC refcodes: (**A**) TEMWUG; (**B)** IFOFUF; (**C)** NUVXAF; (**D)** MESNOR; (**E**) MESPIN; (**F**) WUQLAY; MW: medium-weak; MS: medium-strong; S: strong. Atom colors: C (gray); N (blue); Oxygen (red); S (yellow); Br (orange); I (purple); Cl (green); F (acid green).

The compounds included in this survey belong to different categories of compounds, such as S-containing heterocycles, thioureas, tetrathiafulvalene derivatives, sulfoxides, thiocyanates, thioethers, and disulfides. Some examples are shown in Figure 1, along with the corresponding CSD reference codes.

**Figure 2:**
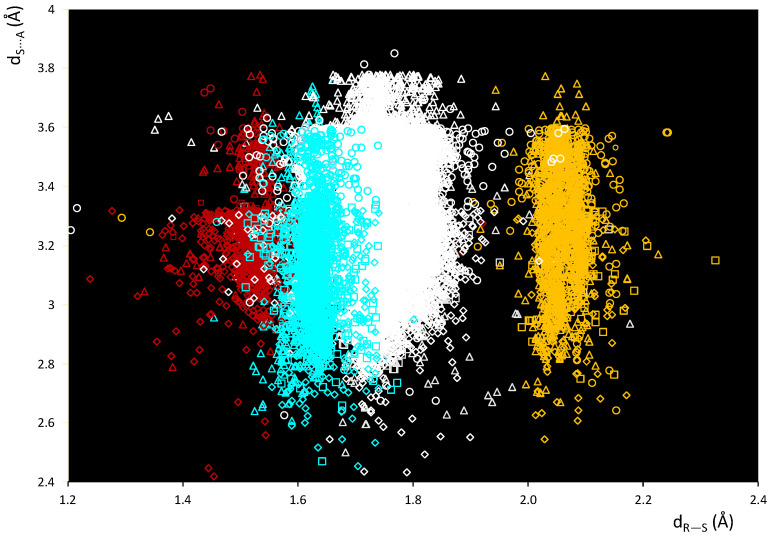
Scatterplot of d_S⋯A_ vs. d_R–S_ distances within fragments R–S⋯A. R = C (white), O (red), N (cyan), and S (yellow); A = N (square), Ch (circle), O (rhomb), and Ha (triangle).

A scatterplot of the d_S⋯A_ and d_R–S_ distances within these fragments is shown in Figure 2. In Figure 2, several areas can be clearly identified corresponding to the different nature of R in R–S⋯A fragments: R = C (white), O (red), N (cyan), and S (yellow). The data fall in regions corresponding to different ranges of d_R–S_ distances at values that can be directly related to the different covalent radii of the species covalently bound to the sulfur atom. Differences can also be highlighted with respect to the van der Waals radius of the interacting atom of the ChB acceptor A (Figure 2 and Figure S1).

**Figure 3:**
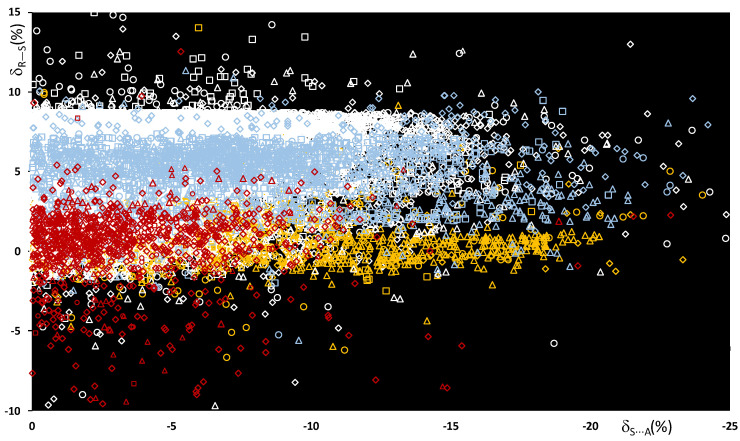
Scatterplot of the δ_R–S_ vs. δ_S⋯A_ functions calculated for all fragments R–S⋯A. R = C (white), O (red), N (light blue), and S (yellow); A = N (square), Ch (circle), O (rhomb), and Ha (triangle).

The bond distances d_R–S_ and ChB interaction distances d_S⋯A_ were used to calculate the functions δ_R−Ch_ and δ_Ch⋯A_ defined below (see Methodology section), and the scatterplot of the values calculated for all R–S⋯A fragments is shown in Figure 3.

In Figure 3, the color code represents the nature of the R atom, and the shape represents the nature of the A ChB acceptors, grouped by category. The examination of the normalized data shows that the relative R–S stretching distances δ_R–S_ do not depend on the atomic radii of the atoms involved, but on the type of atom directly bound to the central sulfur. It can be noted that the functions δ_C–S_ and δ_N–S_, calculated for systems with either a C–S or an N–S covalent bond, fall within a range of 0–9%. On the contrary, when either an O–S or an S–S bond is present, smaller δ_R–S_ values are found, and δ_O–S_ and δ_R–S_ are mainly mainly in the range of −2% to 2%.

An analysis of the $\delta_{S\cdot \cdot \cdot A}$ values allows the strengths of the ChBs in the R–S⋯A fragments to be evaluated and compared. The strength of the S⋯A interaction is indeed directly related to the interaction energy and not to the structural distances. However, several theoretical studies correlate interaction energies and key geometric parameters and show that stronger interactions usually correspond to reduced intermolecular S⋯A distances [30,36,37], and hence to more negative δ_S⋯A_ values. For all the systems examined, the δ_S⋯A_ values range from 0% to −25%, with 88% being between 0% and −15%. It is interesting to note that although examples of the different R–S⋯A categories can be found all over the range, the region corresponding to shorter interactions ($\delta_{S\cdot \cdot \cdot A}\;$< −15%) is mostly populated by fragments with N–S and S–S bonds, whereas fragments with an O–S bond fall mainly in the region of long interactions, with $\delta_{S\cdot \cdot \cdot A}$ between 0% and −10%.

These results agree very well with the recent study by P.A. Wood et al. on σ-hole interactions in small molecules containing divalent sulfur groups R_1_–S–R_2_ [38]. The scatterplot does not show a clear role for the acceptor A, although ionic systems in which the ChBs involve A = Br^−^ and I^−^ as the ChB acceptors show Ch⋯A interaction distances remarkably shorter than the sum of the relevant vdW radii.

**Figure 4:**
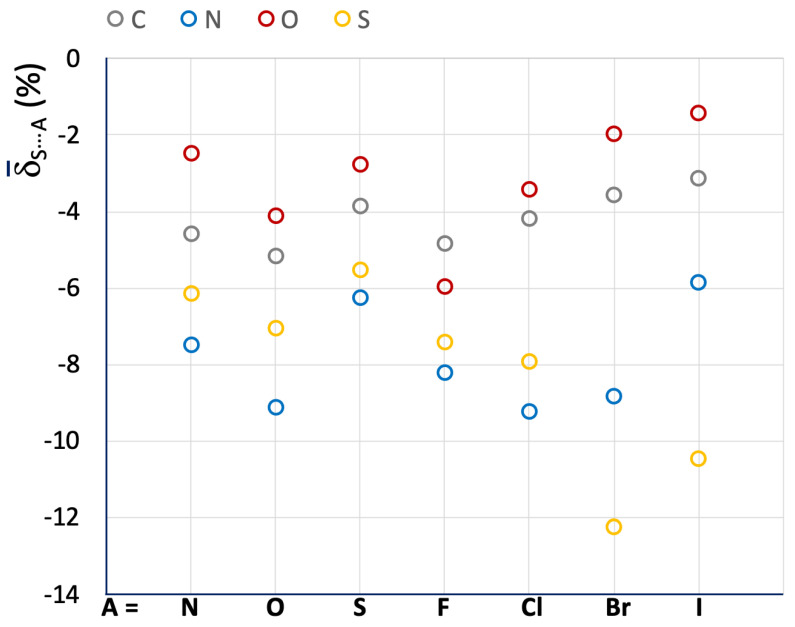
Mean $\bar{\delta }$_S⋯A_ values calculated for R–S⋯A fragments. A = N, O, S, F, Cl, Br, and I; R = C (grey), N (blue), O (red), and S (yellow).

The relative variations were used to calculate the mean $\bar{\delta }$_S⋯A_ values for the different categories of R–S⋯A fragments (R = C, N, O, and S; A = N, O, S, F, Cl, Br, and I), and the resulting data are presented in Table 1 and Figure 4 (color code: R = C (grey), N (blue), O (red), S (yellow)).

Despite the wide variety of systems included, the mean $\bar{\delta }$_S⋯A_ values show significant results. The shortening of the Ch⋯A interaction distance, which can be related to the strength of the relative ChB, increases in the order of O < C < S < N, except when A is a soft halide species (Br^−^ and I^−^). On the contrary, there is no clear trend regarding the role of A. The role of A is likely to be closely related to the chemical nature of the interacting molecules and the overall charge distribution, making it difficult to isolate a general contribution deriving from the nature of the sole atom A. For example, the systems belonging to the C–S⋯A categories show very similar $\bar{\delta }$_S⋯A_ values ranging from –3.13 to –5.15 for A = I and O, respectively, while for the fragments belonging to the S–S⋯A category the $\bar{\delta }$_S⋯A_ values range from –5.54 to –12.25 for A = S and Br, respectively. In any case, it can be noted that the fragments involved in S⋯S interactions (A = S) show small relative shortenings (i.e., less negative $\bar{\delta }$_S⋯A_ values), proving that S⋯S ChBs interactions are usually weak.

In Table 1, the corresponding normalized contact Nc parameter, defined as the ratio of the crystallographic distance between the interacting Ch and A atoms to the sum of their respective van der Waals radii (d_Ch⋯A_)/(r_vdW_Ch + r_vdW_A) [17], is also given for the investigated systems. Nc is very useful for correlating Ch⋯A interaction distances of systems containing different Ch atoms. However, since the shortening of the d_Ch⋯A_ distances with respect to the sum of the vdW radii is not very large, the calculated Nc values usually fall within a narrow range when comparing similar systems. For example, the R–S⋯N fragments parsed in Table 1 show Nc values ranging from 0.93 (R = N) to 0.97 (R = O). On the contrary, the δ_S⋯A_ function is much more sensitive, with mean $\bar{\delta }$_S⋯A_ values ranging from –2.46 (R = O) to –7.48 (R = N) for the same R–S⋯N category of fragments (Table 1 and Figure 4).

### 2.2. R−Se⋯A Fragments

On passing from sulfur to selenium, the number of linear fragments R–Se⋯A results significantly decreased to a total of 4,109 items (see Table S2).

The distribution is similar to that found for the lighter congener, with an evident prevalence (91.4%) of systems with R = C, N, O, S, and Se. In particular, the number of fragments with a S–Se bond is quite low, indicating that the Ch–Ch⋯A fragments belong mainly to homoatomic dichalcogenides. The contribution of all the other systems, which represents 8.6% of the total fragments, is very heterogeneous and it will not be discussed here.

**Figure 5:**
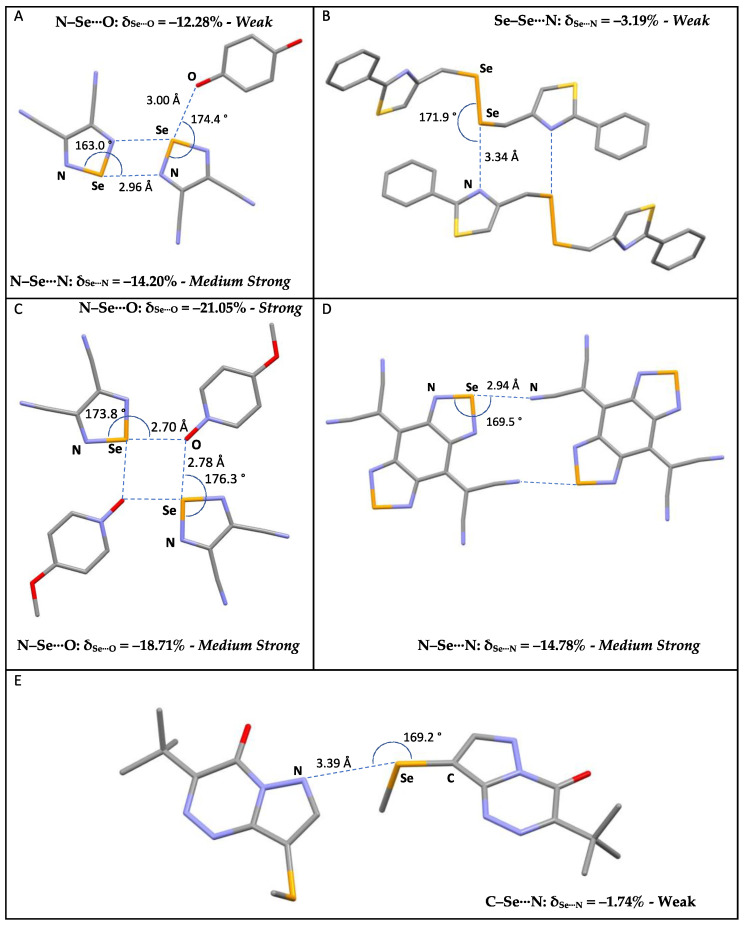
Examples of selected R–Se⋯A fragments featuring a chalcogen bond and corresponding CCDC refcodes: (**A**) GESSEG; (**B**) FEYBAP; (**C**) SAJPAY; (**D**) GEFVOC10; (**E**) XUBFOS01. MW: medium-weak; MS: medium-strong; S: strong. Atom colors: C (gray); N (blue); Oxygen (red); S (yellow); Se (orange).

The compounds investigated are mainly the Se-analogues of those previously studied for the sulfur fragments (Figure 5). This similarity is reflected in the distribution of the d_R–Se_ vs. d_Se⋯A_ distances (Figure S3), which is similar to that observed for the fragments with R–S as the ChB donor. As expected, the d_R–Se_ and d_Se⋯A_ distances involved are longer than those found for the sulfur fragments. A wider dispersion of the values can be observed, especially for the C–Se⋯Ha category of systems, possibly related to the larger polarizability of Se with respect to S.

**Figure 6:**
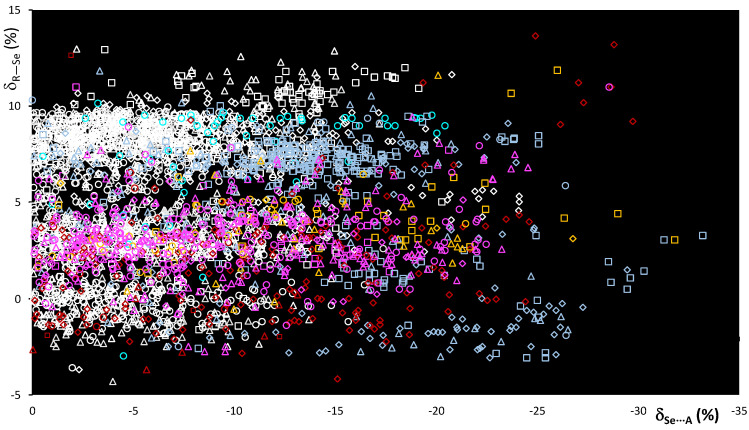
Scatterplot of the δ_R–Se_ vs. δ_Se⋯A_ functions calculated for fragments R–Se⋯A. R = C (white), O (red), N (light blue), S (yellow), and Se (magenta); A = N (square), Ch (circle), O (rhomb), and Ha (triangle).

On passing from sulfur to selenium, despite the different nature and dimension of the atoms involved, the δ_R−Se_ and δ_Se⋯A_ relative distances are comparable, reflecting the similar nature of the systems considered. Accordingly, the scatterplot shown in Figure 6 is comparable to that shown in Figure 3 for the R–S⋯A fragments. Although relative variations δ_R−Se_ fall within the same range of values, a wider dispersion of the fragments can be observed, possibly related to the greater polarizability of Se with respect to S noted above. The δ_Se⋯A_ values reach −30% of relative shortening, and a number of entries, corresponding to 14% of the fragments examined, show values shorter than −15%. This clearly indicates that selenium fragments are involved in stronger ChBs than those found in R–S⋯A fragments (see Figure 3).

**Figure 7:**
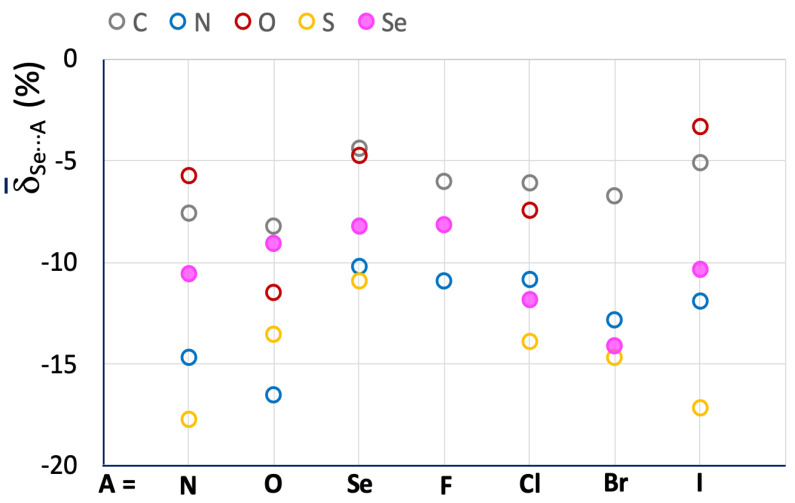
Mean $\bar{\delta }$_Se⋯A_ values calculated for R–Se⋯A fragments. A = N, O, Se, F, Cl, Br, and I; R = C (grey), N (blue), O (red), S (yellow), and Se (filled magenta circles).

The influence of the R atom bonded to the selenium ChB donor is evident, and although each R–Se⋯A category presents values falling all over the range, the region corresponding to shorter interactions ($\delta_{\mathrm{Se}\cdot \cdot \cdot A}$ < –20%) is mostly populated by fragments featuring N–Se bonds. The increase in strength is better demonstrated by the mean $\bar{\delta }$_Se⋯A_ values calculated for the R–Se⋯A fragments (R = C, N, O, S, and Se; A = N, O, Se, F, Cl, Br, and I; Figure 7 and Table 2).

A comparison of the mean$\;\bar{\delta }$_Se⋯A_ and $\bar{\delta }$_S⋯A_ values calculated for the investigated systems (Figure 4 and Figure 7, and Table 1 and Table 2) confirms a significant increase in the strength of ChBs found in R–Se⋯A fragments. Taking the R–Ch⋯N category as an example, values ranging from –5.74 (R = O) to –14.65 (R = N) are found for $\bar{\delta }$_Se⋯A_, with a relative shortening almost double that found for R–S⋯N fragments (Table 1).

The influence of the nature of the R atom directly bonded to the chalcogen atom is slightly different from that found for the lighter congener: the C–Se⋯A and O–Se⋯A fragments confirm small relative shortenings, while the S–Se⋯A fragments are usually involved in interactions with $\bar{\delta }$_Se⋯A_ values shorter than those found for the N–Se⋯A ones, so that the order is O < C < N < S (except for A = O). As noted above for the R–S⋯A fragments, the influence of the nature of the A acceptor is unclear, except for those with A = Se, which show particularly less negative $\bar{\delta }$_Se⋯A_ values, corresponding to weak Se⋯Se ChBs interactions, analogous to the S⋯S ones discussed above.

### 2.3. R−Te⋯A Fragments

**Figure 8:**
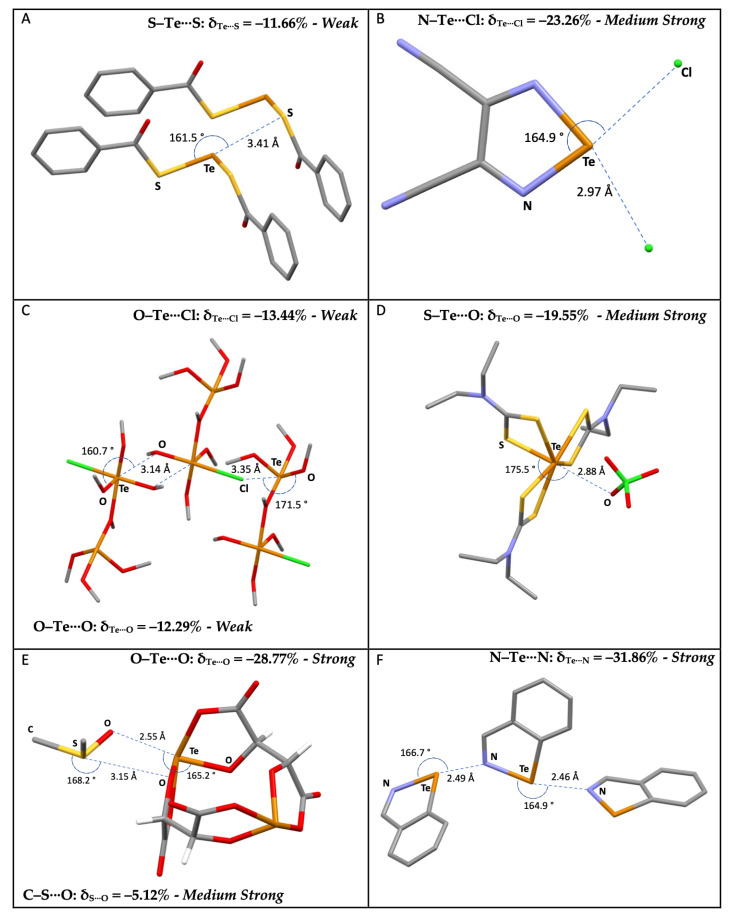
Examples of selected R–Te⋯A fragments featuring ChBs and corresponding CCDC refcodes: (**A**) CISPUP; (**B**) GESSAC; (**C**) QAYVAN; (**D**) JAWPOM; (**E**) NIVRAM; (**F**) BZITEZ. MW: medium-weak; MS: medium-strong; S: strong. Atom colors: C (gray); N (blue); Oxygen (red); S (yellow); Te (orange); I (purple); Cl (green); F (acid green).

Table S3 shows the data for the 2,318 R–Te⋯A fragments deposited at the CSD, categorized according to the nature of the R and A atoms (R = B, C, N, P, As, O, S, Se, and Te; A = N, P, As, Sb, O, S, Se, Te, F, Cl, Br, and I). For the sake of comparison, the analysis was limited to the same categories of fragments investigated for Ch = S and Se (R = C, N, O, S, Se, and Te; A = N, Ch, O, and Ha), which represent 97% of the occurrences. Fragments with R = Te were also considered to include ditellurides, analogous to disulfides and diselenides discussed above. Examples of R–Te⋯A fragments are represented in Figure 8.

**Figure 9:**
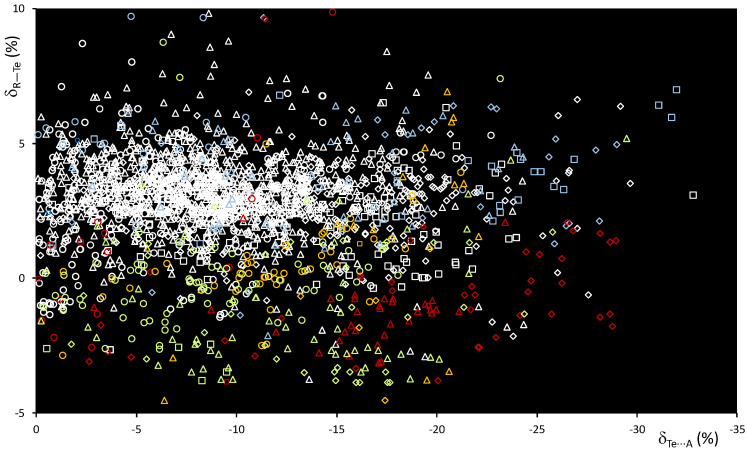
Scatterplot of the δ_R–Te_ vs. δ_Te⋯A_ functions calculated for fragments R–Te⋯A. R = C (white), O (red), N (light blue), S (yellow), Se (magenta), and Te (acid green); A = N (square), O (rhomb), Ch (circle), and Ha (triangle).

The scatterplot of d_Te⋯A_ vs. d_R–Te_ distances (Figure S4) shows a wide dispersion of the values, especially for A = Ha, as previously observed for the R–Se⋯A systems. The scatterplot of δ_R–Te_ vs. δ_Te⋯A_ shows smaller δ_R–Te_ variations than those recorded for the lighter S and Se congeners (Figure 9), with values strongly dependent on the nature of the atoms directly bound to tellurium.

The δ_R–Te_ values indicate unaffected or slightly elongated bond distances for systems with R = C (white), N (light blue), and S (yellow), and shortening of the R–Te distances in fragments with R = Te (acid green) and O (red). At the same time, an increased shortening of the δ_Te⋯A_ values is recorded, with 23% of the fragments exceeding −15% of the relative shortening and several values exceeding the threshold of −30% found for the analogous Se fragments—see, for example, compound F in Figure 8, confirming a strengthening of the ChB interactions involved on passing from R–Se to R–Te donors (Figure 6 and Figure 9).

**Figure 10:**
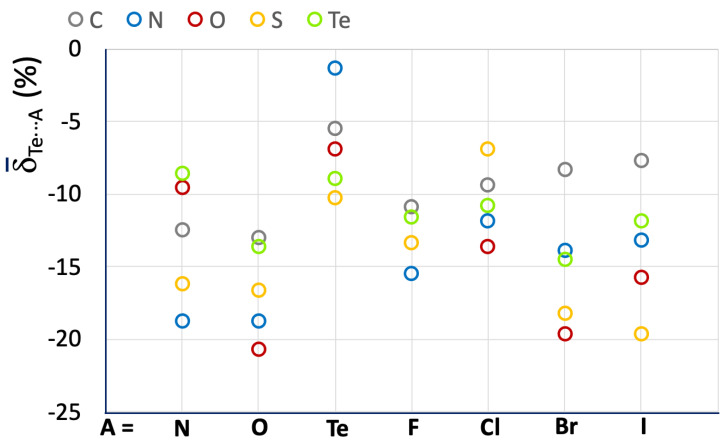
Mean $\bar{\delta }$_Te⋯A_ values calculated for the R–Te⋯A fragments. A = N, O, Te, F, Cl, Br, and I; R = C (grey), N (blue), O (red), S (yellow), and Te (acid green).

The mean values $\bar{\delta }$_Te⋯A_ reported in Figure 10 and Table 3 for the R–Te⋯A fragments indicate an increase in the strength of the related ChBs as compared with the R–Se⋯A systems.

A less regular trend is found as compared to those previously discussed, possibly due to the presence of a smaller number of fragments belonging to more diverse categories. However, some observations on the influence of the nature of the R atom directly bonded to the chalcogen atom can be drawn, confirming that systems with N–Te and S–Te covalent bonds are usually involved in stronger ChBs. Furthermore, fragments with an O–Te covalent bond, usually corresponding to polymeric tellurium oxides (red circles in Figure 10) are often involved in strong ChBs. This feature differs from the trends found for O–S⋯A and O–Se⋯A fragments, which usually show the weakest ChBs. There is no clear trend regarding a dependence on the nature of the ChB acceptor A. However, it can be noted that when A = Te, less negative mean $\bar{\delta }$_Te⋯A_ values are found, corresponding to weaker Te⋯Te ChBs interactions, in agreement with what was observed above for S- and Se-containing fragments.

### 2.4. R–Ch⋯A Fragments: A Scale for ChB Strength

**Figure 11:**
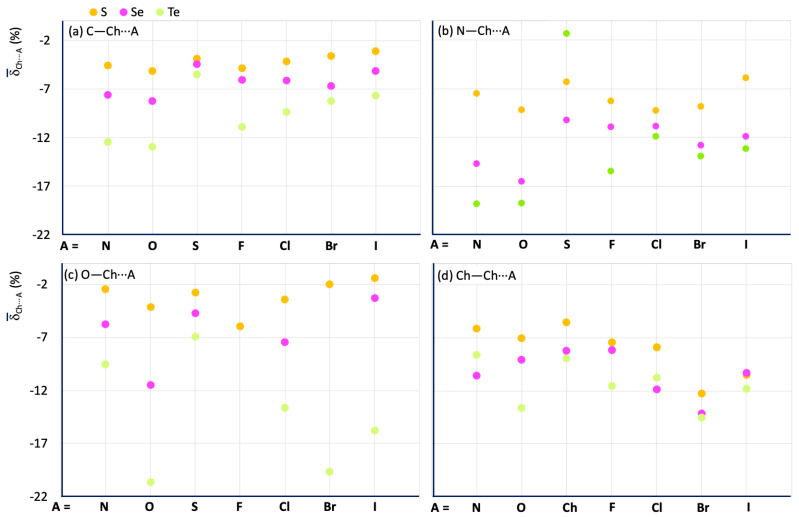
Mean ${\bar{\delta }}_{\mathrm{Ch}\cdot \cdot \cdot A}$ values calculated for the R–Ch⋯A fragments. R = C (**a**), N (**b**), O (**c**), and Ch (i.e., S for S, Se for Se, and Te for Te) (**d**) for Ch = S (yellow), Se (magenta), and Te (acid green).

Figure 11 shows a comparison of the mean $\bar{\delta }$_Ch⋯A_ values calculated for the fragments R–Ch⋯A (Ch = S, Se, and Te; R = C (Figure 11a), N (Figure 11b), O (Figure 11c), and Ch (Figure 11d)). A clear increase in ChB strength is observed on passing from S to Se and Te. The influence of the nature of R confirms a strengthening of ChB interactions in the order N > Ch > C > O, except for the O–Te⋯O and O–Te⋯Ha fragments, as mentioned above (see green dots in Figure 11c). There is no regular variation of the $\bar{\delta }$_Ch⋯A_ values with A for the different R–Ch⋯A categories. However, some conclusions can be drawn: (1) for all the R–Ch⋯A categories, the smaller $\bar{\delta }$_Ch⋯A_ shortenings are associated with A = S, Se, and Te for Ch = S, Se, and Te, respectively; (2) for R = C, and N, the strongest interactions, in terms of delta shortenings, are associated with A = N, and O, for all the examined categories; 3) for R = O, and Ch, strong interactions are found for A = Br, and I; O–Te⋯O interactions are found to be on average stronger than the others in which the tellurium chalcogen is involved.

As shown by the data discussed above (Table 1, Table 2 and Table 3), the delta parameters are remarkably sensitive to the chemical nature of the interacting tectons, and they allow comparison of the ChB interactions in R–Ch⋯A systems with different Ch atoms and discrimination between categories with different R atoms. This is particularly useful when the shortening of the d_Ch⋯A_ with respect to the sum of the vdW radii is modest. A formulation of a ChB strength scale is possible based on the comparison of the δ_Ch⋯A_ values calculated for all the structural data analyzed. In order to also take into account the dependence of $\bar{\delta }$_Ch⋯A_ on the nature of A, the $\bar{\delta }$_av(Ch⋯A)_ value for all A acceptors was calculated for each R–Ch⋯A fragment category (R = C, N, O, and S; Table 4) and the data used to scale the ChB strength.

**Figure 12:**
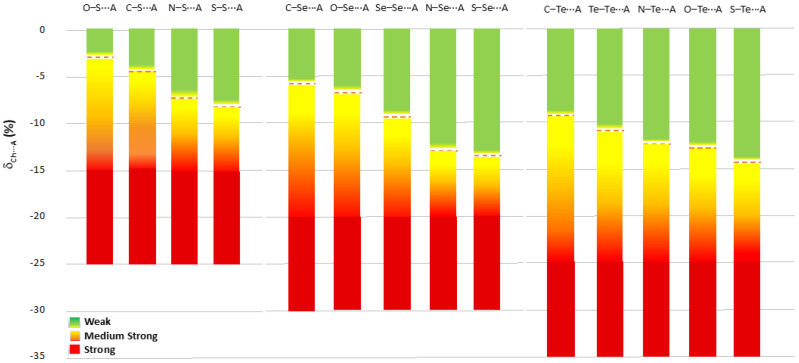
Scale of ChB strength modulated on the nature of the R and Ch species involved in the R–Ch⋯A chalcogen bond ordered for δ_Ch⋯A_. $\bar{\delta }$_av(Ch⋯A)_ values reported as red dotted lines for each different R–Ch⋯A category.

Chalcogen bonds found in R–Ch⋯A fragments with δ_Ch⋯A_ values less negative than the $\bar{\delta }$_av(Ch⋯A)_ value calculated for that specific R–Ch⋯A category (dotted line in Figure 12) can be considered as weak (green in Figure 12); those with δ_Ch⋯A_ values more negative than the $\bar{\delta }$_av(Ch⋯A)_ value can be considered as medium-strong (yellow to red in Figure 12). The ChB can be defined as strong if the relevant δ_Ch⋯A_ parameter exceeds the cutoff values of −15%, −20%, and −25% found for Ch = S, Se, and Te, respectively (red in Figure 12). These cutoff values were chosen by rounding up the lowest mean $\bar{\delta }$_Ch⋯A_ values calculated for the different categories of compounds (Table 1, Table 2 and Table 3). It is worth noting that to date, while strong ChBs are usually evident and easily recognized, a fine categorization has not been reported for interactions with intermediate and weak ChB strengths. The proposed scale allows for a differentiation of the interactions falling in the range between the sum of the vdW radii and the cutoff values defined above. Since this scale is modulated by the nature of the R and Ch species directly involved in the R–Ch⋯A interactions, it allows the ChB strength to be assigned by distinguishing between interactions that have the same relative shortening (with respect to the sum of the relevant vdW radii) but fall in different strength ranges.

## 3. Methodology

To better rationalize the role of the species involved, we performed a systematic search in the Cambridge Structural Database (CSD). CSD searches were performed using ConQuest Version 2022.1.0 on R–Ch⋯A fragments, by imposing an R–Ch⋯A angle between 160 and 180° and a Ch⋯A distance shorter than the sum of the vdW radii involved. R represents the atom directly bound to the chalcogen by a covalent R–Ch bond (Ch = S, Se, and Te), and was allowed to be R = B, C, N, P, As, Sb, O, S, Se, Te, F, Cl, Br, and I. A is the atom directly interacting with the chalcogen and was searched by imposing A = N, P, As, Sb, O, S, Se, Te, F, Cl, Br, and I. The search yielded 34,190 R−Ch⋯A linear fragments, with abundances of 81% (27,763), 12% (4,109), and 7% (2,318) for Ch = S, Se, and Te, respectively (Tables S1–S3).

Sorting the fragments according to the type of species involved (Tables S1–S3) revealed a net numerical prevalence of the R–Ch⋯A systems with Ch = S, Se, and Te; R = C, N, O, S, Se, and Te, and A = N, O, S, Se, Te, F, Cl, Br, and I. Therefore, we decided to limit the analysis to these systems, which covered 97.7%, 91.4%, and 98.8% of the linear R–Ch⋯A fragments deposited for Ch = S, Se, and Te, respectively.

A first analysis of the data was made by directly comparing the d_R–Ch_ bond distances with the d_Ch⋯A_ interaction distances within the R−Ch⋯A fragments, which directly reflect the dimensions of the atoms involved (see Figure 2 and Figures S1–S3 in the Supplementary Material). Therefore, to compare the structural data of R−Ch⋯A fragments involving different atoms, a normalization of the data was required. The structural data were normalized for the relevant covalent and vdW radii (*r_cov_* and *r_vdW_*, respectively) by introducing the functions δ_R−Ch_ and δ_Ch⋯A_ calculated according to Equations (1) and (2):

(1) $$\delta_{R–\mathrm{Ch}}=\frac{d_{R–\mathrm{Ch}}-\left(r_{\mathrm{cov}}R+r_{\mathrm{cov}}\mathrm{Ch}\right)}{\left(r_{\mathrm{cov}}R+r_{\mathrm{cov}}\mathrm{Ch}\right)}\cdot 100$$

(2) $$\delta_{\mathrm{Ch}\cdot \cdot \cdot A}=\frac{d_{\mathrm{Ch}\cdot \cdot \cdot A}-\left(r_{\mathrm{vdW}}\mathrm{Ch}+r_{\mathrm{vdW}}A\right)}{\left(r_{\mathrm{vdW}}\mathrm{Ch}+r_{\mathrm{vdW}}A\right)}\cdot 100$$

The function δ_R−Ch_ represents the relative variation of the d_R–Ch_ bond distance and is independent of the different sizes of the atomic species involved. In a previous paper, this function was exploited to directly compare the relative elongations (δ) of the two bonds in strictly related X–Y–Z three-body systems involving either halogens or halogen and chalcogen atoms [39]. The function δ_Ch⋯A_, referring to the Ch⋯A interaction, was introduced here as a natural extension of the previous definition. Specific single/double-bond covalent radii for the atoms involved in R–Ch bonds have been used depending on the nature of the bond in the fragments considered [10,11].

## 4. Conclusions

The analysis of all the structural data deposited at the CSD made it possible to validate the assumptions generally accepted for ChB interactions, based on either experimental or theoretical studies carried out on different classes of compounds. A scale for evaluating the strength of ChB was developed based on the experimental structural data available for the fragment involved. The study performed on the structural data pertaining to linear R−Ch⋯A fragments featuring a ChB confirms a strengthening in the Ch⋯A interactions with the increase in the polarizability of the chalcogen atom (Te > Se > S), with Ch⋯A interacting distances in the ranges 2.4–3.6, 2.4–3.8, and 2.4–4.1 Å for Ch = S, Se, and Te, respectively (Figure 2, Figures S2 and S3). The increase in the strength of the relevant ChBs can be better appreciated using the functions δ_R−Ch_ and δ_Ch⋯A_ (Equations (1) and (2)), which represent the normalized variation of the bonding d_R–Ch_ distance with respect to the sum of the relevant covalent radii, and the normalized differences between the d_Ch⋯A_ distances and the sum of the relevant vdW radii, respectively.

The calculated δ_Ch⋯A_ values for the R−Ch⋯A fragments show a relative shortening with respect to the sum of the vdW radii involved, increasing in the order Te > Se > S, corresponding to an increase in the ChB interaction strength along the same direction. In fact, although a continuum of values can be found for all R–Ch⋯A fragments involved, the range of δ_Ch⋯A_ values varies from 0% to −20%, −30%, and −35% for Ch = S, Se, and Te, respectively. The nature of the R atom directly bonded to the chalcogen indicates a general strengthening of the ChB interactions in the order R = N > Ch > C > O. No systematic variation of the $\bar{\delta }$_Ch⋯A_ values with A was found for the different R-Ch⋯A categories, even if weak ChBs were found in R–Ch⋯Ch fragments where the chalcogen interacts with its homologous.

The mean $\bar{\delta }$_av(Ch⋯A)_ values calculated for the fragments confirm the same trends, and allow the proposal of a new, sensitive ChB strength scale for R–Ch⋯A fragments: chalcogen bonds with δ_Ch⋯A_ values less negative than $\bar{\delta }$_av(Ch⋯A)_ calculated for this specific category can be defined as weak; those with δ_Ch⋯A_ values more negative than $\bar{\delta }$_av(Ch⋯A)_ can be defined as medium-strong; and those with a shortening greater than −15% for Ch = S, −20% for Ch = Se, and −25% for Ch = Te can be defined as strong.

The definitions used in the proposed ChB strength scale can be applied with remarkable ease and versatility to a wide variety of chalcogen-bonded systems. The proposed scale will also allow researchers to accurately assess the relative strength of the ChB interactions of interacting tectons R–Ch and A, based solely on experimental structural data.

## Figures and Tables

**Table 1:** Mean of δ_S⋯A_^a^ values ($\bar{\delta }$ _S⋯A_) and normalized contact Nc ^b^ values calculated for the fragments R–S⋯A. R = C, N, O, and S; A = N, O, S, Se, F, Cl, Br, and  I.

R–S⋯A	N	O	S	Se	F	Cl	Br	I
**C** $\bar{\delta }$_S⋯A_ Nc	−4.57 0.95	−5.15 0.95	−3.87 0.96	−4.69 0.95	−4.83 0.95	−4.18 0.96	−3.58 0.96	−3.13 0.97
**N** $\bar{\delta }$_S⋯A_ Nc	−7.48 0.93	−9.13 0.91	−6.25 0.94	−4.18 0.96	−8.22 0.92	−9.24 0.91	−8.81 0.91	−5.85 0.94
**O** $\bar{\delta }$_S⋯A_ Nc	−2.46 0.97	−4.12 0.96	−2.77 0.97	−2.12 0.98	−5.95 0.94	−3.41 0.97	−1.97 0.98	−1.41 0.99
**S** $\bar{\delta }$_S⋯A_ Nc	−6.14 0.94	−7.05 0.97	−5.54 0.94	−8.87 0.91	−7.42 0.93	−7.92 0.92	−12.25 0.88	−10.47 0.89

^a^ $\delta_{S\cdots A}=\frac{d_{S\cdots A}-\left(r_{\mathrm{vdW}}S+r_{\mathrm{vdW}}A\right)}{\left(r_{\mathrm{vdW}}S+r_{\mathrm{vdW}}A\right)}\cdot 100$; ^b^ $\mathrm{Nc}=\frac{d_{S\cdots A}}{\left(r_{\mathrm{vdW}}S+r_{\mathrm{vdW}}A\right)}$.

**Table 2:** Mean of δ_Se⋯A_^a^ values ($\bar{\delta }$ _Se⋯A_) and Nc^b^ values calculated for the fragments R–Se⋯A. R = C, N, O, S, and Se; A = N, O, S, Se, Te, F, Cl, Br, and I.

R–Se⋯A	N	O	S	Se	Te	F	Cl	Br	I
**C** $\bar{\delta }$_Se⋯A_ Nc	−7.6 0.92	−8.2 0.92	−4.2 0.96	−4.4 0.96	−3.6 0.96	−6.03 0.94	−6.12 0.94	−6.71 0.93	−5.11 0.95
**N** $\bar{\delta }$_Se⋯A_ Nc	−14.65 0.85	−16.5 0.83	−14.2 0.86	−10.2 0.90	−	−10.88 0.89	−10.85 0.89	−12.8 0.87	−11.9 0.88
**O** $\bar{\delta }$_Se⋯A_ Nc	−5.74 0.94	−11.45 0.88	−	−4.71 0.95	−	−	−7.45 0.92	−	−3.30 0.97
**S** $\bar{\delta }$_Se⋯A_ Nc	−17.71 0.82	−13.56 0.86	−6.91 0.93	−10.93 0.89	−	−	−13.87 0.86	−14.69 0.85	−17.15 0.83
**Se** $\bar{\delta }$_Se⋯A_ Nc	−10.56 0.89	−9.05 0.91	−11.21 0.89	−8.21 0.92	−0.43 0.99	−8.13 0.92	−11.84 0.88	−14.12 0.86	−10.31 0.90

^a^ $\delta_{\mathrm{Se}\cdot \cdot \cdot A}=\frac{d_{\mathrm{Se}\cdot \cdot \cdot A}-\left(r_{\mathrm{vdW}}\mathrm{Se}+r_{\mathrm{vdW}}A\right)}{\left(r_{\mathrm{vdW}}\mathrm{Se}+r_{\mathrm{vdW}}A\right)}\cdot 100$; ^b^ $\mathrm{Nc}=\frac{d_{\mathrm{Se}\cdot \cdot \cdot A}}{\left(r_{\mathrm{vdW}}\mathrm{Se}+r_{\mathrm{vdW}}A\right)}$.

**Table 3:** Mean of δ_Te⋯A_^a^ values ($\bar{\delta }$ _Te⋯A_) and Nc^b^ values calculated for the fragments R–Te⋯A. R = C, N, O, S, Se, and Te; A = N, O, S, Se, Te, F, Cl, Br, and I.

R–Te⋯A	N	O	S	Se	Te	F	Cl	Br	I
**C** $\bar{\delta }$_Te⋯A_ Nc	−12.46 0.87	−12.96 0.87	−7.73 0.92	−9.03 0.91	−5.46 0.94	−10.89 0.89	−9.39 0.91	−8.25 0.92	−7.69 0.92
**N** $\bar{\delta }$_Te⋯A_ Nc	−18.77 0.81	−18.71 0.81	−5.71 0.94	−	−1.33 0.99	−15.44 0.85	−11.84 0.89	−13.91 0.86	−13.13 0.87
**O** $\bar{\delta }$_Te⋯A_ Nc	−9.53 0.90	−20,67 0.79	−2.69 0.97	−	−6.91 0.97	−	−13.61 0.86	−19.67 0.80	−15.76 0.84
**S** $\bar{\delta }$_Te⋯A_ Nc	−16.15 0.84	−16.66 0.83	−12.2 0.88	−	−10.27 0.90	−13.34 0.87	−6.86 0.93	−18.26 0.82	−19.64 0.84
**Se** $\bar{\delta }$_Te⋯A_ Nc	−8.58 0.91	−13.61 0.86	−5.93 0.94	−	−8.92 0.91	−11.55 0.88	−10.78 0.89	−14.51 0.85	−11.81 0.88

^a^$\;\delta_{\mathrm{Te}\cdot \cdot \cdot A}=\frac{d_{\mathrm{Te}\cdot \cdot \cdot A}-\left(r_{\mathrm{vdW}}\mathrm{Te}+r_{\mathrm{vdW}}A\right)}{\left(r_{\mathrm{vdW}}\mathrm{Te}+r_{\mathrm{vdW}}A\right)}\cdot 100$; ^b^ $\mathrm{Nc}=\frac{d_{\mathrm{Te}\cdot \cdot \cdot A}}{\left(r_{\mathrm{vdW}}\mathrm{Te}+r_{\mathrm{vdW}}A\right)}$.

**Table 4:** Average $\bar{\delta }$ _av(Ch⋯A)_ value of the mean $\bar{\delta }$ _Ch⋯A_ values calculated for all the A acceptors in the fragments R–Ch⋯A. R = C, N, O, S, Se, and Te; A = N, O, S, Se, Te, F, Cl, Br, and I.

$\bar{\delta }$ **_av(S_** **_⋯A)_**	C–Ch⋯A	N–Ch⋯A	O–Ch⋯A	S–Ch⋯A	Se–Ch⋯A	Te–Ch⋯A
**Ch = S** $\bar{\delta }$ **_av(S_** **_⋯A)_**	−4.41	−7.40	−3.07	−8.21	−	−
**Ch = Se** $\bar{\delta }$ **_av(Se_** **_⋯_** **_A)_**	−5.77	−12.75	−6.53	−13.55	−9.32	−
**Ch = Te** $\bar{\delta }$ **_av(Te_** **_⋯A)_**	−9.32	−12.36	−12.69	−14.17	−	−10.71

## Data Availability

The data were retrieved from the Cambridge Structural Database (CSD) using ConQuest Version 2022.1.0. The searches were performed on QA–Ch⋯QB fragments by imposing a QA–Ch⋯QB angle between 160 and 180°. Ch⋯QB distances shorter than the sum of the relevant van der Waals radii were imposed. QA = B, C, N, P, As, Sb, O, S, Se, Te, F, Cl, Br, and I; QB = N, P, As, Sb, O, S, Se, Te, F, Cl, Br, and I; Ch = S, Se, and Te. The data resulting from the research were elaborated using Microsoft Excel version 16.52, 2021, and reported in the present work as graphs and tables.

## Supplementary Materials

The following supporting information can be downloaded at https://www.mdpi.com/article/10.3390/molecules28073133/s1: Scheme S1: Perturbation molecular orbital scheme showing the σ-interaction between the ChB acceptor A and the σ*_R−Ch_ orbital of the ChB donor fragment; Figure S1: Scatterplot of the d_Ch⋯A_ vs. d_C—Ch_ distances within the fragments C–Ch⋯A.; Figure S2: Scatterplot of the δ_Ch⋯A_ vs. δ_C—Ch_ values calculated for the fragments C–S⋯A.; Figure S3: Scatterplot of d_Se⋯A_ vs. d_R—Se_ distances within the fragments R–Se⋯A.; Figure S4: Scatterplot of the d_Te⋯A_ vs. d_R—Te_ distances within the fragments R–Te⋯A. Tables S1–S3: Occurrence of the linear fragments R−S⋯A, R−Se⋯A, and R−Te⋯A.
