# Supplementary material for: A Structural Approach to the Strength Evaluation of Linear Chalcogen Bonds
Source: Molecules. 2023 Mar 31;28(7):3133. doi: 10.3390/molecules28073133 (PMC10096081; doi:10.3390/molecules28073133)
Supplement: Supplementary file 1 [file molecules-28-03133-s001.zip › SI_molecules-2195274_final.pdf]

SUPPLEMENTARY MATERIAL

A structural approach to the strength evaluation of linear chalcogen bonds

M. Carla Aragoni [1]*, Massimiliano Arca [1], Vito Lippolis [1], Anna Pintus [1], Yury Torubaev [2], Enrico Podda [1,3]

[1] Dipartimento di Scienze Chimiche e Geologiche, Cittadella Universitaria Monserrato, SS. 554 – bivio Sestu, 09042 Monserrato, Cagliari, Italy

[2] Department of Chemistry, Ben-Gurion University of the Negev, 84105 Beer-Sheva, Israel

[3] Centro Servizi di Ateneo per la Ricerca, Università degli Studi di Cagliari, Cittadella Universitaria, S.S. 554 bivio Sestu, 09042 Monserrato, Cagliari, Italy.

*Correspondence: aragoni@unica.it

Table of Contents

Scheme S1: Perturbation molecular orbital scheme showing the σ-interaction between the ChB acceptor A and the $\sigma^*_{R\text{–}Ch}$ orbital of the ChB donor fragment (right). S2

Figure S1: Scatterplot of the $d_{Ch\cdots A}$ *vs* $d_{C—Ch}$ distances within the fragments C—Ch···A. S3

Figure S2: Scatterplot of the $d_{Ch\cdots A}$ *vs* $d_{C—Ch}$ values calculated for the fragments C—S···A. S4

Figure S3: Scatterplot of $d_{Se\cdots A}$ *vs* $d_{R—Se}$ distances within the fragments R—Se···A. S5

Figure S4: Scatterplot of the $d_{Te\cdots A}$ *vs* $d_{R—Te}$ distances within the fragments R—Te···A. S5

Table S1: Occurrence of the linear 27763 fragments R–S···A featuring a ChB sorted by the nature of the involved atoms. S6

Table S2: Occurrence of the linear 4109 fragments R–Se···A featuring a ChB sorted by the nature of the involved atoms. S6

Table S3: Occurrence of the linear 2318 fragments R–Te···A featuring a ChB sorted by the nature of the involved atoms. S7

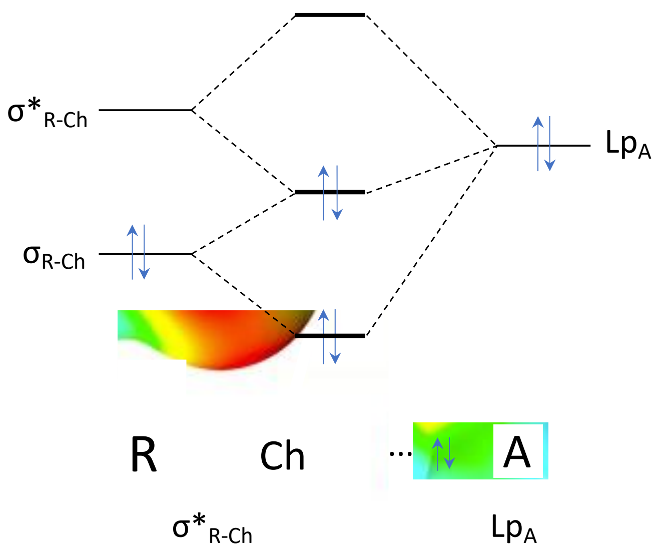

Scheme S1: Perturbation molecular orbital scheme showing the σ-interaction between the ChB acceptor A and the σ^*_{R-Ch} orbital of the ChB donor fragment (right).

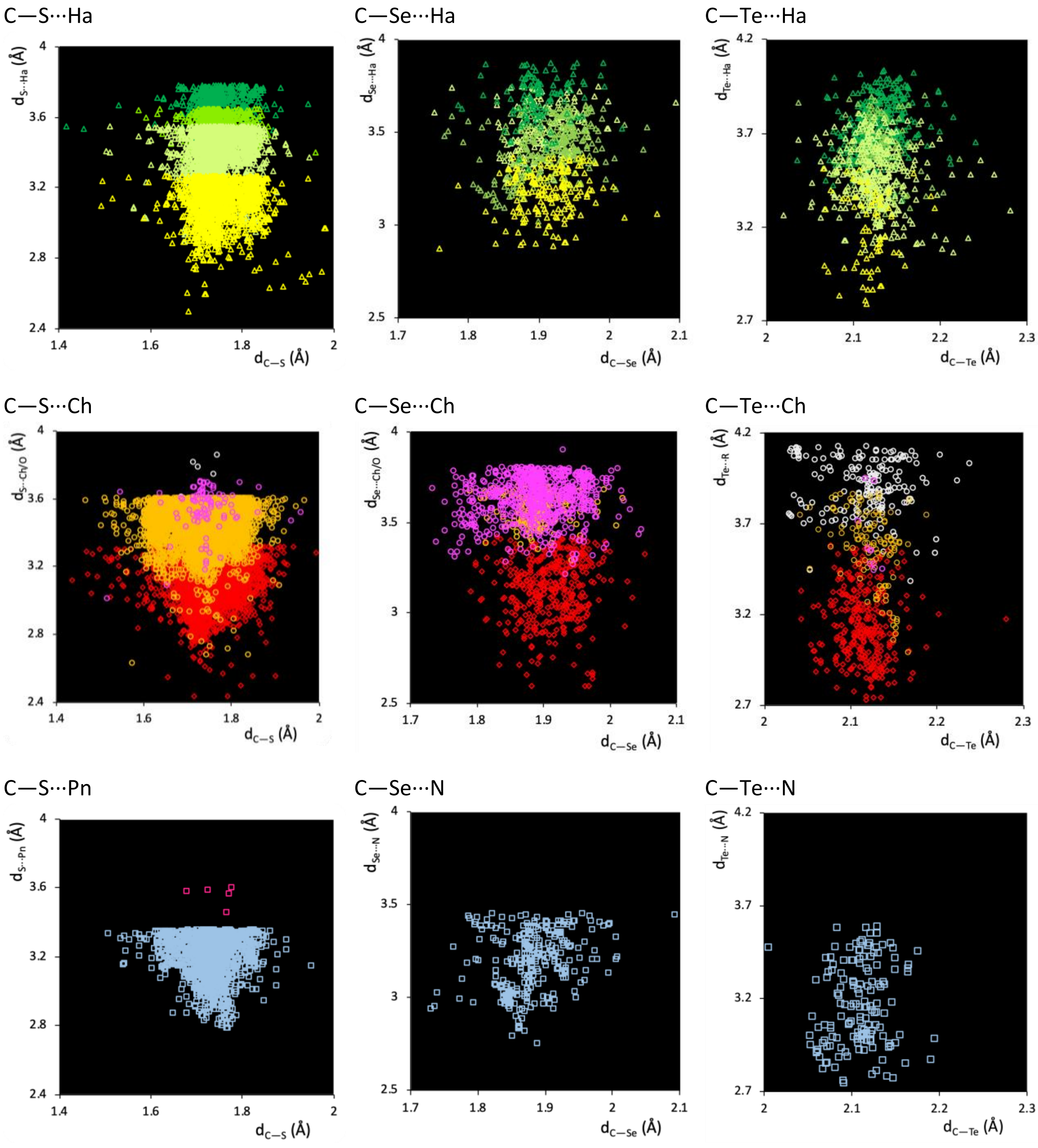

Figure S1: Scatterplot of the $d_{Ch\cdots A}$ *vs* $d_{C—Ch}$ distances within the fragments C—Ch···A. A = Ha [triangles: F (lemon yellow), Cl (light green), Br (apple green), I (dark green)]; Ch/O [Ch = circles: S (yellow), Se (magenta), Te (white); O = rhombs (red)]; Pn [squares: N (light blue) P (purple)].

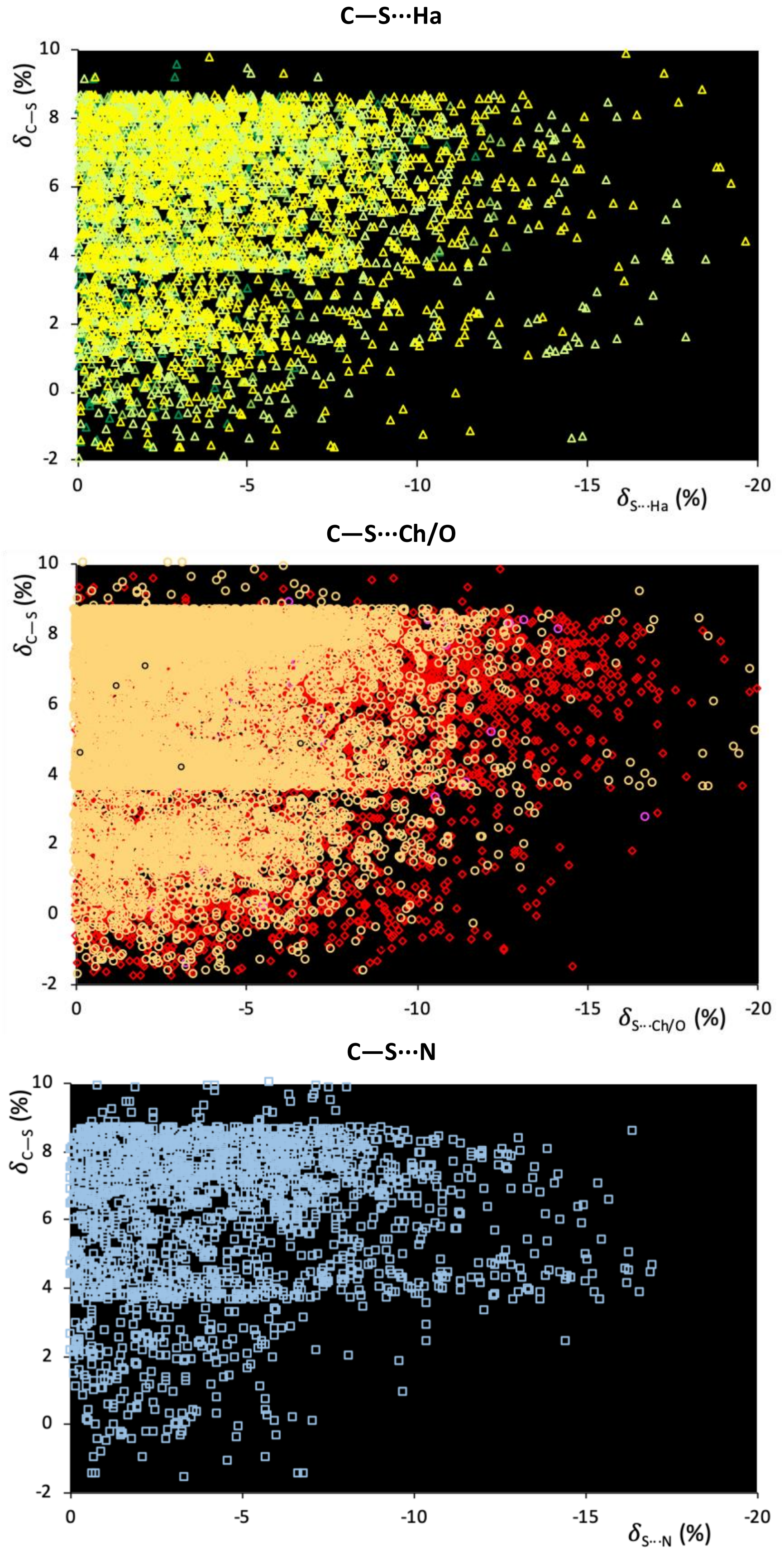

Figure S2: Scatterplots of the $d_{Ch\cdots A}$ *vs* $d_{C—Ch}$ values calculated for the fragments C—S···A. A = Ha [triangles: F (lemon yellow), Cl (light green), Br (apple green), I (dark green)]; Ch/O [Ch = circles: S (yellow), Se (magenta), Te (white); O = rhombs (red)]; N (light blue squares).

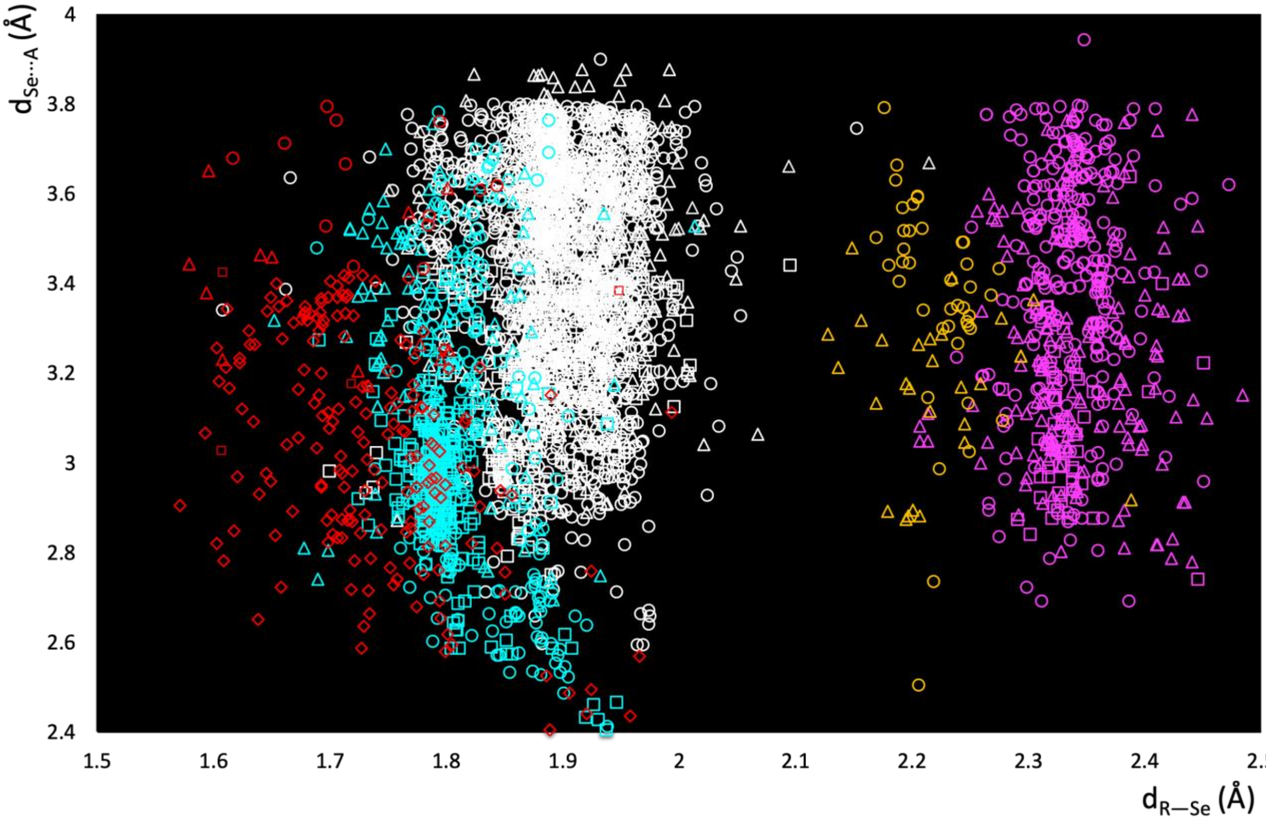

Figure S3: Scatterplot of $d_{Se\cdots A}$ *vs* $d_{R—Se}$ distances within the fragments R—Se···A. R = C (white), O (red), N (cyano), S (yellow); Se (magenta); A = N (square), Ch (circle), O (rhombus), Ha (triangle).

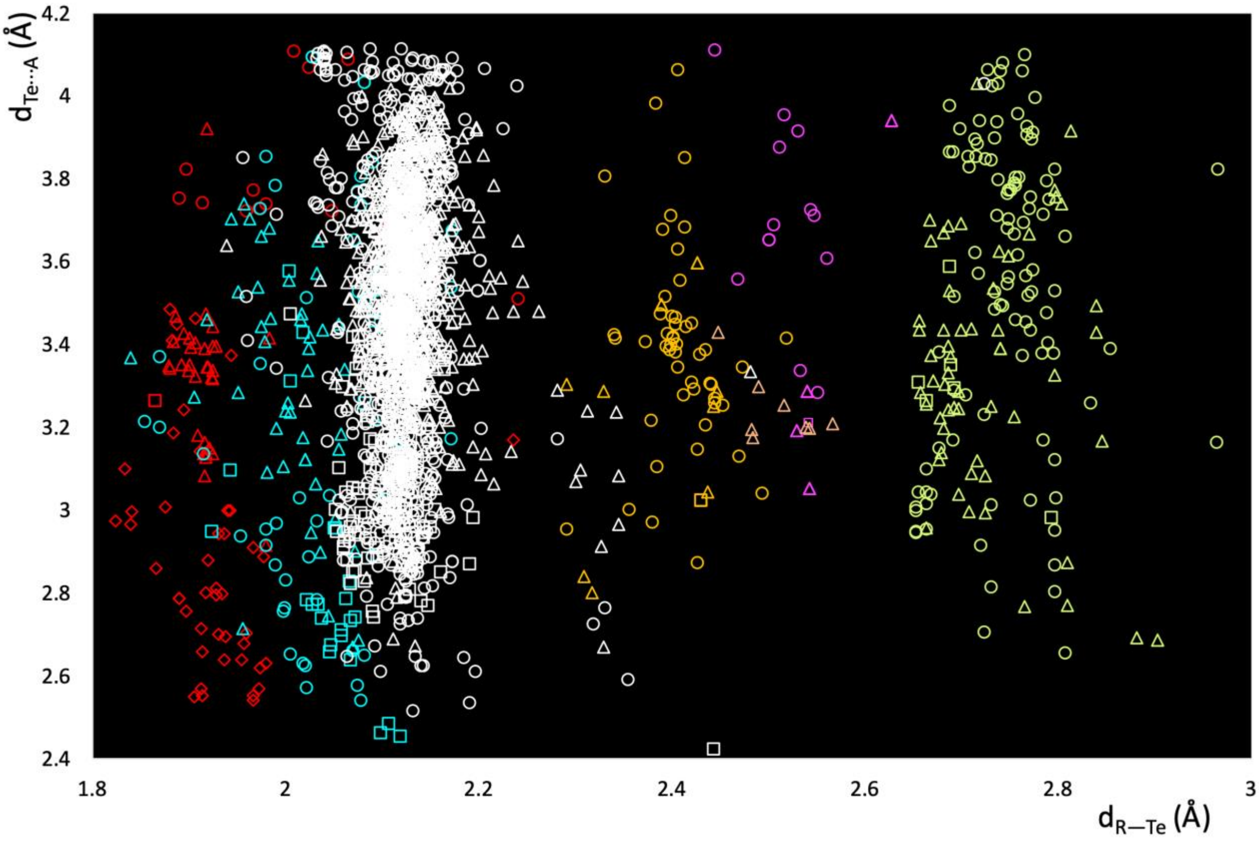

Figure S4: Scatterplot of the $d_{Te\cdots A}$ *vs* $d_{R—Te}$ distances within the fragments R—Te···A. R = C (white), O (red), N (cyano), S (yellow); Se (magenta); Te (acid green). A = N (square), Ch (circle), O (rhomb), Ha (triangle).

Table S1: Occurrence of the linear 27763 fragments R–S···A featuring a ChB sorted by the nature of the involved atoms. R = B, C, Si, N, P, As, Sb, O, S, Se, Te, F, Cl, Br, and I; A = N, P, As, Sb, O, S, Se, Te, F, Cl, Br, and I.

**R–S···A**	**N**	**P**	**As**	**Sb**	**O**	**S**	**Se**	**Te**	**F**	**Cl**	**Br**	**I**
**B**	0	0	0	0	5	2	0	0	6	9	0	0
**C**	1975	5	0	1	6537	8784	87	9	1511	1871	661	552
**Si**	4	0	0	0	0	1	0	0	0	0	0	0
**N**	1116	0	0	0	363	341	3	0	244	297	27	35
**P**	6	1	0	0	17	163	2	1	12	23	5	1
**As**	0	0	12	0	1	24	0	0	1	12	0	1
**Sb**	11	0	0	91	0	32	0	0	0	3	0	0
**O**	36	0	0	0	836	29	2	2	71	101	18	6
**S**	180	0	0	0	199	642	4	0	145	177	186	69
**Se**	5	0	0	0	1	2	8	0	0	12	12	1
**Te**	0	0	0	0	1	6	1	4	1	3	3	2
**F**	13	0	0	0	22	0	0	0	59	0	0	0
**Cl**	2	0	0	0	4	1	0	0	10	11	0	0
**Br**	0	0	0	0	0	0	0	0	1	0	6	0
**I**	0	0	0	0	0	1	0	0	0	0	1	2

Table S2: Occurrence of the linear 4109 fragments R–Se···A featuring a ChB sorted by the nature of the involved atoms. R = B, C, Si, N, P, As, Sb, O, S, Se, Te, F, Cl, Br, and I; A = N, P, As, Sb, O, S, Se, Te, F, Cl, Br, and I.

**R–Se···A**	**N**	**P**	**As**	**Sb**	**O**	**S**	**Se**	**Te**	**F**	**Cl**	**Br**	**I**
**B**	0	0	0	0	0	0	1	0	0	0	1	0
**C**	272	1	0	0	424	186	718	1	171	292	182	116
**Si**	0	0	0	0	0	0	1	0	0	0	0	0
**N**	253	0	0	0	118	11	57	0	28	70	13	23
**P**	1	9	0	0	9	4	141	1	9	15	5	21
**As**	3	0	3	0	1	0	11	0	0	0	0	0
**Sb**	6	0	0	1	0	0	4	0	0	0	0	0
**O**	4	0	0	0	189	0	13	0	0	13	0	1
**S**	28	0	0	0	5	20	16	0	0	19	5	4
**Se**	54	1	1	0	44	26	209	1	41	46	48	33
**Te**	0	0	0	0	0	0	1	3	2	3	2	1
**F**	0	0	0	0	0	0	0	0	2	0	0	0
**Cl**	5	0	0	0	6	0	2	0	23	32	0	0
**Br**	0	0	0	0	0	0	1	0	0	0	16	0
**I**	0	0	0	0	0	0	1	0	0	1	0	4

Table S3: Occurrence of the linear 2318 fragments R–Te···A featuring a ChB sorted by the nature of the involved atoms. R = B, C, Si, N, P, As, Sb, O, S, Se, Te, F, Cl, Br, and I; A = N, P, As, Sb, O, S, Se, Te, F, Cl, Br, and I.

R–Te···A	N	P	As	Sb	O	S	Se	Te	F	Cl	Br	I
**B**	0	0	0	0	1	0	0	0	3	0	0	1
**C**	155	1	0	0	332	127	10	185	66	352	165	376
**Si**	0	0	0	0	0	0	0	1	0	0	0	0
**N**	28	0	0	0	28	14	0	2	13	23	3	9
**P**	0	2	0	0	3	2	0	9	0	3	4	5
**As**	0	0	0	0	3	1	0	0	0	0	0	0
**Sb**	0	0	0	2	0	0	0	0	0	0	0	0
**O**	1	0	0	0	50	7	0	7	0	4	8	21
**S**	1	0	0	0	5	38	0	7	4	2	3	8
**Se**	1	0	0	0	0	1	3	8	1	1	1	1
**Te**	6	0	0	1	34	4	0	67	20	13	10	17
**F**	0	0	0	0	0	0	0	0	0	0	0	0
**Cl**	1	0	0	0	3	0	0	1	1	9	0	0
**Br**	0	0	0	0	1	0	0	0	2	0	16	0
**I**	0	0	0	0	0	0	0	0	1	3	0	12
